# What Is the Simillimum?

**Published:** 1890-01

**Authors:** M. A. A. Wolff

**Affiliations:** Gainesville, Tex.


### WHAT IS THE SIMILLIMUM?

### (And maybe Some Other Stray Questions.)

M. A. A. Wolff, M. D., Gainesville, Tex.

April 9th, 1889, called to Mrs. J. I found her in bed very prostrated, coughing. All I am now able to state is that I gave her *Lyc.*$^{1400}$, one dose, placebo, and *Puls.*$^{200}$, the last one to be taken, if needed, on account of night cough. My second call was on April 14th, and my prescription Sil.$^{72000}$. On April 30th Mr. J. came to pay his bill, and gave a glorious report. A few months later her mother (dispenser of homœopathic benefits, and, as she stated, agent for Micayah's Uterine Wafers) visited me before leaving for the North, and spoke very pleasantly of her daughter's condition. I then gave her Dr. Rogers' symptom-book.

December 27th.—Mr. J. brought me a statement from his wife, who cannot be induced personally to see a physician or permit any examination, but who would willingly consult in writing, however, only with me. The first part of the statement was but the number of groups of symptoms. The number of medicines these characteristic symptoms called for was twenty-three. Out of these, nineteen had only one, two, three, or four symptoms, the rest, Bell. seven, Rhus-t. seven, Lycop. eight, and Sepia nine. Arranging these symptoms as seen below according to our materia medica, and studying the four medicines I found Sepia to cover exactly thirty-five symptoms, and many of the rest by implication, analogy, or synonymic expressions. The symptoms are: "Easily tired out, restless, continually changing position, cannot at night get easy in any position or lie still a moment. Restless, easy in each new position but for a moment (Rhus-tox.). Always in a hurry, but accomplishes little. Cries very easily. Great tendency to start. Headache affects exactly half of the head. Intense aching in back of head and nape of neck. Boring pain in right side of head. Head hot, feet cold. Dark circles around the eyes, which are sunken. Light dim, as looking through a fog. Blurring sight, with falling of the womb. Black spots hovering and swimming before the eyes. Thin, watery, burning discharge from the eyes. Pale face and lips, with great debility. The complexion has a greenish hue. Grayish yellow color of face. Feels at times as if a ball were rising in throat. Weak digestion, the simplest food disagrees. Craves salt, likes food very salty. Awakens at night very hungry. Feels as if there were a band around the waist. Much rumbling *left* side of abdomen. Walls of abdomen sore. Falling out of bowel from moderate straining at stool. Great bearing down in abdomen and back. Stool hard, difficult, seems to slip back. Rectum seems not to have power to expel stool. Urine scanty, dark. Frequent desire to urinate. Urine escapes on coughing, sneezing, blowing the nose. Red sand-like sediment in urine. Urine covered with greasy scum on standing. Yellow urine, looking like saffron. Cannot bear even pressure of clothing over womb. Great bearing down in region of womb; displacement, falling of womb; feeling that she must cross legs lest womb fall out. Pain in groin and womb, worse from slightest jar. Breast very painful through every monthly period. Menses too often, come every two to three weeks. Great weakness after menses. Menses profuse, lasting from five to ten days. Pimples on skin worse during menstruation. Palpitation of heart, cannot lie on back. The least motion causes palpitation of heart. An indescribable feeling about heart. Heart trouble, with numbness of left arm and shoulder. Backache, relieved by lying flat on back. Great bearing-down pain in back and abdomen. Bruised pain at lower end of spine. Severe pain through hips and in back. Great weakness of small of back and legs. Feet cold and clammy on removing stockings. Troubled much with coldness of knees. One foot cold while other warm. Hands and feet go to sleep easily. Numbness and paralytic feeling of legs. Feels stiff in rising from seat. Lumbago, severe aching of back. Icy coldness of feet. Dreams of great exertion, wakens exhausted. Feels well on rising, but gives out by noon. Pains come and go quickly. Sitting up an hour late is greatly felt next day. Upper part of body much thinner than lower part. Ill effects from overexertion. Bad effects from straining and lifting."

I expect that the taking down of these symptoms must have been for several days. But she concludes her list by stating: "Suffer sharp, darting pains in top part of the womb which only last a few minutes. Womb so low at times that it comes out on stooping down, and it can be seen with a mirror. Around the mouth of the womb are small granulations like grains of sugar and the neck presents a purple-red color. Suffer faintness at times, also during menstration, after twelve hours stops entirely for twelve or eighteen hours—suffering like child-birth until it starts again with a great gush, when relieved until well." Prescribed Sepia.$^{200}$, to be taken at once, plenty placebos, and Sepia$^{1600}$ if relapse. At the same time gave prepared sponge and glycerole of Calendula in water,—the sponge to be used only when on her feet (during day).

January 1st.—Mr. J. stated that she had only used one powder (Sep.$^{200}$) and delivered the following report:

"First day after using treatment felt very badly in the afternoon and did not sleep much all night. Had feeling in head *resembling* dizziness, or as if I were falling, and severe pain low down in back."

"Second day I felt very comfortable and worked around on my feet nearly all day, but could not rest or sleep at night much."

"Third day did not feel so well again, and had a great deal of water pass from the womb every time I made any exertion of a straining kind, like rising from a stooping position, or sneezing or coughing, etc.; felt very nervous and irritable and oversensitive to noises. None of the time have I suffered any *severe* pain since the first day, but an uneasy, nervous feeling some of the time yesterday; felt as if menses were on only not severe. Will have them Sunday, if regular, would feel much better if able to sleep."

Six powders, Puls.$^{200}$, for bed-time, *if needed.*

Jan. 7th, 5.30 P. M., received the following report: "Menses appeared on time, Sunday, Jan. 5th, much as usual. I do not suffer the first few hours (say about ten hours), after that I do. This time I commenced to have quite severe pain after nine hours, which took the form of labor-pains. They grew very hard about nine o'clock that night, and kept increasing until about 1.30 Monday morning, when I fell into the first sleep, and was much easier after that. Each pain brought a discharge of blood, but not profuse. After it began coming profusely the pains subsided, although they kept up all day Monday, until about ten o'clock at night. I was *quite* hot with fever till nearly morning (Tuesday). I felt too weak to rise and go about my duties. My head aches severely in the left side, down to the end of my nose. I slept very well after taking a sleeping-powder [Puls.$^{200}$], but am very sore across me where the pains were, and weak, as though everything would fall out, and give way in back and abdomen. I was not suffering any *more* than usual, nor so long as I usually dŏ, but I feel *utterly prostrated.* About one year ago I took a severe cold, and coughed a great deal, which always hurts me more in the lower part of the abdomen and lungs. I had for nearly two years before that suffered at my periods with stoppages. When my cough was so hard I strained myself, which, I suppose, caused my menses to come two weeks too soon and *profuse*, and a stoppage, after which great prostration, from which I could not rally until time for another similar 'spell.' This kept up until the strange attack the last of February, when you came to see me. [She is mistaken, it was April, as stated above.] I have never been well since. I got up from that sickness very weak, and with frequent faintings. Most of the time I have worn an electric or magnetic belt, low down across the region of the womb, as a support, as it seemed without it I could not keep myself together. While at Hot Springs I had a painless period, which only lasted four days, and it was an epoch in my life, for I never passed through with more than three or four easy periods in my life. Mother told a physician in Chicago about me, and he told her if I would take one drop of the tincture of Ergot for one week, then skip a week, etc., it would help me; that my womb was flabby [how did he know?], and did not contract. I tried it one week, and my period came on (too soon), and, though painful, no stoppage. The next month I took it again, and it came six days too soon, with labor-pains and no stoppage, but too profuse. Have not taken it for a month now, but had about the same kind of time. I have always got up and assumed my duties when I would have done better to have been idle, but I have a horror of being an invalid, or always complaining of myself while my husband is at home, so I always put the best side out to him, and don't tell him much of how I feel, unless I look badly and he asks me. Then I have often felt so badly that I could hardly keep up, and when he comes I seem to feel better (whether it is the joy and happiness of having him with me, or the influence of magnetism about him, I cannot tell), but after he goes I feel gradually let down, just as the effect of a stimulant leaves any one. His presence has always affected me in this way. I feel less pain when he rubs me, or lays his hand on me. I think I am very easily affected. I have written quite lengthy, but thought it might acquaint you with my state of health and temperament."

The messenger left me about half an hour, when, after having studied Dysmenorrhœa treatment in Guernsey, I decided on sending *Carb. anim.*$^{3000}$. At 6.30 P. M. he received it with orders if his mother were not better in two hours to return, else to come in the morning, January 8th. He did not return before the morning. The powder had acted well, she felt much better, had slept good, but had sweat profusely. [Was that a pathogenetic effect?]

I sent this morning Cimicifuga third, one drop three times a day till next period. Did I do wrong?

